# Room for improvement in non-pharmacological systemic sclerosis care? — a cross-sectional online survey of 650 patients

**DOI:** 10.1186/s41927-020-00142-7

**Published:** 2020-07-31

**Authors:** Juliane K. Stöcker, Madelon C. Vonk, Frank H. J. van den Hoogen, Maria W. G. Nijhuis-van der Sanden, Julia Spierings, J. Bart Staal, Ton Satink, Cornelia H. M. van den Ende, on behalf of the ARCH study group

**Affiliations:** 1 Department of Rheumatology, Sint Maartenskliniek, Nijmegen, The Netherlands; 2 Musculoskeletal Rehabilitation Research Group, HAN University of Applied Sciences, Nijmegen, The Netherlands; 3 Department of Rheumatology, Radboud University Medical Center, Nijmegen, The Netherlands; 4 Radboud Institute for Health Sciences, IQ Healthcare, Radboud University Medical Center, Nijmegen, The Netherlands; 5 Department of Rheumatology and Clinical Immunology, University Medical Center Utrecht, Utrecht, The Netherlands; 6 Research Group Neuro Rehabilitation, HAN University of Applied Sciences, Nijmegen, The Netherlands; 7 European Masters of Science in Occupational Therapy, HvA University of Applied Sciences, Amsterdam, The Netherlands

**Keywords:** Systemic sclerosis, Health professionals, Health care use, Cross sectional survey

## Abstract

**Background/ objective:**

To gain insight in the use of current systemic sclerosis (SSc) care provided by health professionals from the patient perspective. We focused on referral reasons, treatment goals, the alignment with unmet care needs, and outcome satisfaction.

**Methods:**

Dutch SSc patients from 13 participating rheumatology departments were invited to complete an online survey. Descriptive statistics were used to describe current use of non-pharmacological care and outcome satisfaction. Reasons for referral and treatment goals were encoded in International Classification of Function and Disability (ICF) terms.

**Results:**

We included 650 patients (mean (standard deviation [SD]) age, 59.4 (11.4) years. 50% had contact with a health professional in the past year; 76.3% since disease onset. Physiotherapists were the most frequently visited in the past year (40.0%), followed by dental hygienists (11.4%) and podiatrists (9.2%). The three most common referral reasons were pain, joint mobility and cardiovascular functions. Fatigue, Raynaud’s phenomenon, physical limitations, reduced hand function and joint problems were mentioned by more than 25% of all respondents as unmet needs. The proportion of patients treated in the past year by a health professional who were satisfied with knowledge and expertise of their health professionals was 74.4%; 73% reported improved daily activities and better coping with complaints. However, 48.9% perceived that the collaboration between rheumatologist and health professional was never or only sometimes sufficient.

**Conclusion:**

Despite the high outcome satisfaction and good accessibility of health professionals, there are obstacles in the access to non-pharmacological care and communication barriers between health professionals and rheumatologists.

## Background

Systemic sclerosis (SSc) is an orphan connective tissue disease characterized by progressive fibrosis and vasculopathy affecting the skin and multiple internal organs [1]. Despite a growing body of knowledge and new therapeutic approaches, SSc remains a potentially fatal disease with a high clinical burden [2, 3]. SSc can affect the physical and psychological conditions, daily functioning, and participation in society. Pain, digital ulcers, fatigue, and joint contractures significantly contribute to impaired functional capacity and are associated with negative perceptions of illness severity [4–6]. Depression, distressing appearance transformation, social isolation, and Raynaud’s phenomenon have high impact on health-related quality of life (HRQoL) in patients with SSc [7–9].

In recent years, an increased understanding of the disease and targeted research activities have led to an improved classification and a growing number of pharmacological treatment options for specific complications. Much effort has been made to identify the patients’ perspective on their disease, quality of life and potential therapeutic targets [10–12]. Owing to the direct impact of the disease on daily functioning and psychosocial well-being of patients, non-pharmacological care is a key element of SSc care. So far, the evidence for non-pharmacological approaches in SSc is limited and specific guidelines are not available yet [13]. According to the updated European League Against Rheumatism (EULAR) recommendations, the evaluation of the efficacy of non-pharmacological treatments in SSc is on the research agenda for the next update [14].

Restricted access to trustworthy information, including knowledgeable health professionals, and lack of support in managing difficult social interactions and negative emotions are seen as unmet needs in SSc care [13]. A previous qualitative study among rheumatologists revealed barriers for referral to health professionals due to the lack of evidence for non-pharmacological treatments and a correspondingly low confidence of rheumatologists in health professional competences [15]. In the study of Willems et al. among European health professionals about the content of non-pharmacological care, discrepancies between physicians’ reasons for referral and treatment targets as defined by health professionals were found. This also suggests a fragmented knowledge of physicians about the content of non-pharmacological care and a suboptimal communication between physicians and health professionals [16].

Today, patients have an important role in the organization of their own care [17]. Shared decision making contributes to optimal healthcare for SSc patients in terms of improvement of health outcomes, quality of care, and healthcare services. So far, it has not been investigated how SSc patients value non-pharmacological care, the coordination between rheumatologist and health professional, and to what extent this care fits the patients’ needs. Therefore, it is important to involve the patients’ perspective, as alignment in the communication between the different stakeholders is likely to lead to more effective personalized SSc care.

The purposes of this study were to provide insight in [1] the use of the current SSc care provided by health professionals from the patient perspective. We focused on [1] the use of care [2] referral reasons and treatment goals, [3] their alignment with reported unmet care needs, and [4] outcome satisfaction with health professional.

## Methods

### Design

A multicenter, cross-sectional, online survey was performed to explore health care utilization and perceptions of SSc patients in the Netherlands.

### Participants

In the Netherlands, the Arthritis Research and Collaboration Hub (ARCH) was established as a nationwide effort to improve health care for patients with rare systemic autoimmune diseases, including SSc. The ARCH working group purposely selected the departments of rheumatology for the study, to ensure a representative patient population from both regional (*n* = 7) and university (*n* = 6) hospitals spread across the Netherlands. Patients with a registered diagnosis of SSc, treated in one of the 13 participating rheumatology departments, were selected from the patient administration system of the institution and invited to participate. Information about the survey was communicated to the patients by the treating rheumatologists. The invitation was accompanied by a written participant information letter and a reply card. After returning the reply card or sending a notification e-mail, a unique web link was distributed to enter the online survey. The inclusion criteria were as follows: being diagnosed with SSc, aged ≥18 years, and sufficient knowledge of the Dutch language. Data were processed anonymously. All participants provided informed consent when starting the web survey and before they were asked substantive questions.

Ethical approval was obtained by the Institutional Review Board of the Radboud university medical center, Nijmegen (protocol number: 2017–3621). The Strengthening the Reporting of Observational Studies in Epidemiology (STROBE) guidelines were followed [18].

### Data collection

The online survey was hosted by Castor Electronic Data Capture (Castor EDC; Castor, Amsterdam, the Netherlands), a highly secured, cloud-based electronic data capture platform [19]. The survey questions were constructed based on the results of a literature review, three semi-structured multicenter focus group interviews with 23 patients, and interviews with 12 rheumatologists and five specialized nurses. Next, the survey was evaluated by the members of the ARCH SSc working group and a patient panel [20]. The questionnaire contained 67 multiple choice, multiple response, and open questions covering the following: [1] sociodemographic characteristics; [2] opinions on bottlenecks and areas for improvement; [3] perceived quality of care, and [4] non-pharmacological care. The survey was pilot tested in five SSc patients. To answer the research question of this study, we used data concerning non-pharmacological care and unmet needs in SSc care.

### Description of the selected questions of the survey

#### Sociodemographic questions

Sociodemographic questions included sex, age, educational level, living situation, employment and disability status, and disease characteristics (disease subset, symptom onset and year of diagnose).

#### Unmet needs in SSc care

The question *‘I would like more attention to be paid in my treatment to the following* topics’ was assessed using a list of 27 yes/no questions on changed appearance, physical limitations, pain, fatigue, impaired walking and/or hand function, sleeping problems, psychological problems, sexual dysfunction, stomach and intestine problems, reduced mouth function, gynecological complaints, Raynaud’s phenomenon, joint problems, loss of independence, loss of work / school, daily activities, and social life; insufficient support from social network, dealing with uncertainty, unpredictability of SSc, ambiguities about the diagnosis, feeling misunderstood, loneliness, loss of self-confidence, contact with other SSc patients, and the possibility to indicate other topics.

#### Non-pharmacological care

To assess the use of non-pharmacological care, patients were asked whether they consulted one or more health professionals because of SSc-related problems, since onset of the disease (yes/no) and during the last 12 months (yes/no). Patients who consulted one or more health professionals during the last 12 months were asked to identify the professional most frequently contacted. The list offered included the following health professionals: dietitians, occupational therapists, physiotherapists, hand therapists, speech- and language therapists, social workers, dental hygienists, exercise therapists, podiatrists, and psychologists. Moreover, patients could add other health professional disciplines to the list. Referral reasons and treatment goals were assessed by open-ended questions.

Two subscales, such as “coordination and alignment of care” (four questions) and “your health professional” (three questions) from the Consumer Quality Index (CQI) (rheumatoid arthritis, version 2.0), which has been found to be reliable to measure patients’ experience with the quality of care in the field of rheumatology, were adapted for the current study [21]. Only questions of those two subscales focusing on communication, alignment, and outcome satisfaction with health professional treatments were selected. In addition, the wording “healthcare providers” was changed into “between rheumatologist and health professionals” in 4 questions of the subscale “coordination and alignment of care”. Items were assessed on a four (five)-point Likert-scale [never, sometimes, most of the time, always, (I don’t know)].

### Data analysis

Descriptive statistics were used to describe demographic characteristics, unmet needs, current use of non-pharmacological care, and outcome satisfaction. Statistical analyses were conducted using Stata/IC 13.1 (StataCorp LP, College Station, TX). The free-text responses on the open-ended questions about reasons for referral to HPs and treatment goals were read and re-read to obtain an overview of the collected data. To examine the alignment of referral reasons to unmet needs, the concepts were compiled verbatim and subjected to an exploratory thematic analysis [22]. Coding discrepancies were resolved by discussion between two researchers (JS and CME) before refining the codes by summarizing and encoding in ICF terms (categories and subcategories) using the following:

- the updated ICF linking rules [23];
- the World Health Organization (WHO) ICF browser [24];
- the International Classification of Functioning, Disability and Health (ICF) Core Sets for rheumatoid arthritis [25]; and
- concepts of functioning and health as identified to be important to SSc patients [26].

The proportion of patients with unmet needs was calculated related to the number of patients that reported that need.

## Results

**Fig. 1:**
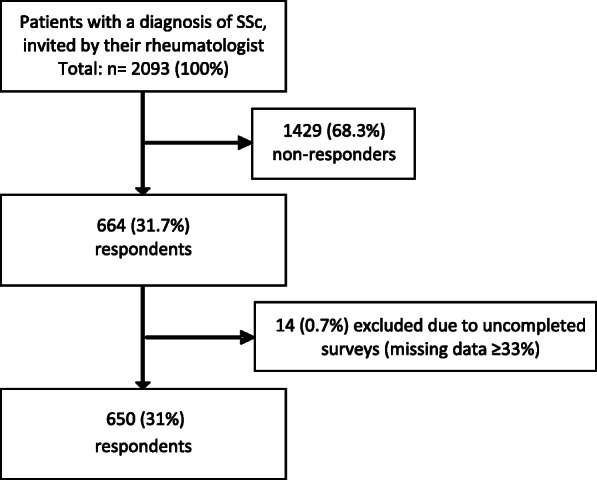
Flow diagram of patient selection procedure

A total of 2093 Dutch patients with SSc were invited to take part in the study from December 15th, 2017 to January 21st, 2018. Among the 2093 invited patients, 664 answered the survey. Data of 14 patients were excluded from the analysis, because of incompleteness. Thus, a total of 650 surveys were included in the analyses (Fig. 1).

Demographics and disease characteristics of the 650 respondents are displayed in Table 1. The majority of the responding patients were women (*N* = 486; 74.8%), with a mean age of 59.4 years (standard deviation [SD] = 11.4) and a mean time since onset of 8.2 years (SD = 8.0). About one third of the respondents received a higher education, and 82% (*N* = 533) were married or living together. Only 37.7% of the respondents were employed.

**Table 1:** Demographic and disease characteristics of 650 patients with SSc

Characteristics	
Female, N (%)	486 (74,8)
Age, years; mean (SD), range	59.4 (11.4), 18–87
Education level, N (%)	
0–12 years	443 (68.2)
> 12 years	207 (31.8)
Living alone, N (%)	117 (18.0)
Paid employment (%)	245 (37.7)
Disease subtype (%)	
Limited	227 (34.9)
Diffuse	132 (20.3)
Subtype unknown	291 (44.8)
Disease duration, years; N (SD), range^a^	8.2 (8.0), 0–51
Mean time between onset and diagnosis, years; N (SD)^a^	4.3 (6.9)
Women	4.8 (5.1)
Men	2.5 (7.3)

^a^Due to missing values, *N* = 646

### Use of care provided by health professionals

Since the onset of disease symptoms (mean time 8.2 years), 469 (76.3%) of the 650 participants had contacted one or more health professionals and half of them (324; 49.9%) had consulted at least one health professional in the last year. Approximately half of these patients (48.8%) were referred by a rheumatologist, a quarter of them (25.9%) contacted health professional themselves. The three most frequently visited health professionals were physiotherapist (40.0%), dental hygienist (11.4%), and podiatrist (9.2%) (Table 2). Approximately three quarters of all patients (76.3%) consulted at least one health professional since SSc onset for SSc-related problems and slightly more than half of these patients (56.6%) had contact with a physiotherapist.

**Table 2:** Health professional uitlization by 650 patients with SSc

	Contacted in last 12 month N (%)	Contacted since onset SSc N(%)
Health professionals (all)	312 (41.1)	469 (76.3)
Physio therapist	260 (40.0)	367 (56.5)
Occupational therapist	58 (8.9)	155 (23.9)
Podiatrist	60 (9.2)	103 (15.9)
Hand therapist	18 (2.8)	58 (8.9)
Exercise therapist	17 (2.6)	28 (4.3)
Dietitian	51 (7.9)	108 (16.6)
Dental hygienist	74 (11.4)	95 (14.6)
Speech therapist	6 (0.9)	22 (3.4)
Psychologist	42 (6.5)	80 (12.3)
Social worker	15 (2.3)	64 (9.9)

### Referral reasons and unmet needs in SSc care

Regarding the open-ended questions about referral reasons and treatment goals, we received a total of 697 encodable responses. We found that patients could not clearly distinguish between referral reasons (reflecting the rheumatologist’s perspective) and treatment goals (reflecting the health professional’s perspective) and consequently gave similar answers to both questions. Therefore, the responses of both questions were combined into one (“referral reasons”) before initial coding. Within these responses, 143 different reasons for referral were identified and subsequently linked to 28 ICF-codes. The most common responses were related to the following ICF categories: pain in body part (38.9%), mobility of joint functions (28.7%), functions of the cardiovascular system (23.1%), functions of the skin and related structures (20.7%), and muscle functions (18.2%). The 15 most frequently mentioned referral reasons are shown in Table 3.

**Table 3:** Top 15 out of 27 different reasons for referral to non-pharmacological care (*N* = 324)

Referral reason (ICF terms)	ICF code	N (%)
Body structures and functions		
Pain in body part	b2801	126 (38.9)
Mobility of joint functions	b710	93 (28.7)
Functions of the cardiovascular system, other specified and unspecified	b429	75 (23.1)
Functions of the skin and related structures, other specified	b898	67 (20.7)
Muscle functions, other specified and unspecified	b749	59 (18.2)
Neuromusculoskeletal and movement related functions	b7	30 (9.3)
Emotional functions	b152	27 (8.3)
Energy and drive functions	b130	16 (4.9)
Weight maintenance functions	b530	15 (4.6)
Respiration functions	b440	12 (3.7)
Blood vessel functions	b415	11 (3.4)
Activities and participation		
Self-care	d5	15 (4.6)
Hand and arm use	d445	34 (10.5)
Moving around in different locations	d460	12 (3.7)
Personal and environmental factors		
Assistive products and technology for personal use in daily living	e1251	11 (3.4)

Fatigue, Raynaud’s phenomenon, physical limitations, reduced hand function, and joint problems were mentioned by more than 25% of all respondents as an unmet need in SSc care (Table 4). An analysis of potential associations of the number of unmet needs with disease duration, age, SSc subtype and education level revealed that participants with a lower level of education have on average 6.4% more unmet needs than participants in the higher educated group.

**Table 4:** Top 5 unmet needs compared to HP treatments aiming the specific unmet need

	More attention to … N (%)	Received non-pharmacological treatment in the last 12 month N (%)	Received treatment aiming at treatment goal related to unmet need N (%)
Fatigue	296 (45.6)	159 (24.5)	15 (5.1)
Raynaud’s phenomenon	205 (31.6)	103 (15.9)	10 (4.9)
Physical limitations	193 (29.7)	119 (18,3)	93 (48.2)
Reduced hand function	177 (27.3)	100 (15.4)	23 (13.0)
Joint problems	163 (25.1)	82 (12.6)	81 (49.7)
No unmet needs	134 (20.7)	does not apply	does not apply

### Alignment of reasons for referral and unmet needs

A relatively small percentage of the respondents (ranging between 4.9 and 13.0%) received non-pharmacological treatment addressing their specific unmet needs. Patients who had not reported any unmet need (20.7%) received a less frequently non-pharmacological treatment (Table 4).

### Coordination and alignment of care

Nearly half of the 324 patients (*N* = 158, 48.9%) who received non-pharmacological treatment in 2017 perceived the collaboration between the rheumatologist and their health professional never or only sometimes as sufficient. Approximately two third of the patients (*N* = 214, 66.2%) reported insufficient agreements between the rheumatologist and the health professional, whereas more than half of the patients (*N* = 162, 50.2%) assumed that the advice given to the patient by the rheumatologist and health professional were never or rarely well-tuned (Table 5).

**Table 5:** Perceived quality of communication between patient, rheumatologist and HP, and outcome satisfaction with HP treatment (*N* = 324)

	Always/mostly	Never/ sometimes	I don’t know
**Perceived quality of communication**			
Did your therapist, after your opinion, collaborate well with your rheumatologist?	56 (17.3)	158 (45.3)	109 (33.8)
How often did your rheumatologist and your therapist, in your opinion, make good agreements with each other?	27 (8.3)	214 (66.2)	82 (25.4)
How often did you think that the treatments and advices you received from your rheumatologist and your therapist were well tuned to each other?	60 (18.5)	162 (50.2)	101 (31.3)
How often was your rheumatologist aware of agreements you had with your therapist?	106 (32.9)	154 (47.7)	63 (19.5)
**Outcome satisfaction**			
Did your therapist have sufficient knowledge and expertise to treat you?^a^	240 (74.3)	36 (11.2)	47 (14.6)
Could you improve your daily activities through the treatment of your therapist?^a^	243 (75.2)	80 (24.8)	does not apply
Can you deal better with your complaints through the treatment of your therapist?^a^	243 (75.2)	80 (24.8)	does not apply

^a^Due to missing values, *N* = 323

### Outcome satisfaction

A total of 240 (74.4%) out of the 324 respondents were satisfied with the knowledge and expertise of their health professionals regarding SSc treatment. The proportion of patients who could cope better with their complaints after the treatment and reported improvement in their daily activities was 73% (*N* = 156) (Table 5).

## Discussion

The results of our study demonstrate that, from the patient’s point of view, the reason for referral to health professionals was primarily the treatment of physical symptoms, such as mobility of joint functions and functions of the cardiovascular system. Reported unmet care needs as fatigue, Raynaud’s phenomenon, and reduced hand function were not strongly covered by the referral reasons. Patients felt satisfied with health professional treatment content and outcomes. Despite this, communication and collaboration between rheumatologists and health professionals were rated rather low, and nearly one third of the patients was not able to judge the quality of communication between their rheumatologist and the health professional.

Our current study has shown that care for people with SSc is not yet optimal. We found three major areas that may be the causes of the different unmet needs for SSc care, which are as follows: underutilization of referrals to HP dealing with the psychosocial aspects of the disease, referrals that are not well aligned to the patients’ unmet needs, and a suboptimal coordination and alignment of care.

### Underutilization of non-pharmacological care services

Only approximately 50% of patients in our study used non-pharmacological care in the last year. Much of the reported referral reasons (reported by more than 30% of the patients) was related to treatment of physical symptoms. Referrals to occupational therapists, psychologists, and social workers, better equipped to address the psycho-social aspects of the disease, including emotional issues, impaired work, and decreased participation in social life, were much rarer [26]. This latter agrees with an earlier study of Willems and suggests that rheumatologists may be more likely to refer to physiotherapists and other HP disciplines who have a focus on the treatment of physical symptoms [16]. This strong focus on referrals to physical treatments possibly reflects obstacles from the following origins: rheumatologists, patients, and lack of evidence. Patients may not be aware enough of the possibilities of the non-pharmacological care. It is also possible that rheumatologists have a lack of knowledge of content and aims of non-pharmacological treatment options [15, 16]. In addition, there is still a lack of strong evidence of the effectiveness of non-pharmacological treatment options [10]. However, since non-pharmacological treatments often do not focus on a specific disease, but rather on symptoms or limitations in activities, evidence for many non-pharmacological treatments originally intended for other rheumatic conditions could also be relevant in this patient group [27]. For instance the evidence for the effectiveness of treatments for commonly SSc specific problems such as fatigue, reduced hand function, and joint problems are already available in other rheumatological diseases [28–30]. HPs should take the opportunity and establish evidence-based recommendations for accessible and targeted non-pharmacological interventions.

### Unmet needs

Along with the low number of referrals for psycho-social reasons, we found a limited alignment between unmet needs and reasons for (self)referral. Especially among patients who identified fatigue and Raynaud’s phenomenon as an unmet care need, only a low percentage reported to actually be treated for this reason. Patients may hesitate to disclose certain topics during the consultation with the rheumatologist and therefore may not discuss their needs for information on non-pharmacological treatment options [14]. A recent study showed that patients with arthritis found it difficult to involve themselves in the decision making, often because they were unaware of having a choice [31]. This supports that the reported unmet care needs are not sufficiently addressed in daily SSc care and suggests that the use of care for SSc patients is still suboptimal. Psycho-social symptoms that are commonly experienced by SSc patients and have a major impact on daily activities and participation need to be considered as primary targets for interventions.

### Coordination and alignment of care

In our study patients perceived the quality of communication and care coordination between rheumatologists and HPs as rather low. Well-coordinated and integrated care is considered as one of the eight important indicators of quality and safety, from the patient perspective [30]. It is not easy to offer SSc patients appropriate and well-coordinated care due to the complexity of the disease, the variability of the disease course, and the limited evidence-supported pharmacological and non-pharmacological treatment options [10, 12, 32].

However, poor communication and coordination, can create additional barriers to care access. SSc patients and their families are feel exposed to great barriers in access to and the quality of specialized and coordinated healthcare [16, 33, 34]. They describe themselves as being “passed around”, have difficulties to find reliable information about their illness and treatment, and experience follow-up appointments logistically, physically and emotionally demanding. This in turn leads to emotional burden and frustration for the patients. This implies that, in daily practice, clinicians must invest even more in the quality of communication, particularly in the promotion of interdisciplinary communication. The use of patient decision aids leads to an increased communication and knowledge, more accurate risk perceptions, and a greater number of decisions consistent with SSc patients’ values, and needs [35]. Our study underlines the importance to develop decision aids that support communication and may lead to decisions more consistent with the patients’ needs.

### Outcome satisfaction

In addition to the three areas of attention, we also found a supporting factor for the use of non-pharmacological care. In this study, patients perceived a high outcome satisfaction with non-pharmacological treatments, as well as high satisfaction with SSc specific knowledge and expertise of health professionals. They experienced improvement of daily activities and symptoms because of the non-pharmacological treatments. As far as we know, this is the first study describing the satisfaction with health professional treatment outcomes in SSc care from the patient perspective in such a large cohort. This underlines the added value of HPs in the treatment of problems that restrict SSc patients in daily activities, although there is not yet much evidence for non-pharmacological treatments.

Regarding our method, some limitations were found that may have influenced the described outcomes or their interpretation. Patients could not clearly distinguish between referral reasons and treatment goals. This might have led to a misinterpretation from the patients’ perspective and made it impossible to distinguish between the rheumatologists’ perspective as reflected in the referral reasons and the health professional treatment goals.

Another limitation of our study might be the relatively large percentage of respondents (58%) that were treated in hospitals specialized in SSc treatment. These patients may have different preferences than patients in small, local hospitals who did not participate.

Third, to recruit a large group of patients, we could only send one invitation without a reminder, which could explain the estimated response rate of 31%. However, the response rate will be slightly higher, as patients treated in shared care (39% of patients) could have received the invitation twice if both centers participated in the study. Compared to previous national and international SSc studies, the composition of our cohort is comparable in terms of demographic and disease specific characteristics. We found two minor differences that we believe do not affect the results of our study; namely large age range of the participants (18–87 years), which is often significantly narrower in comparable studies; and a relatively large percentage of participants, with an unknown SSc subtype (44.8%). However, this percentage is comparable with other surveys classifying patients in subtypes of SSc on the basis of self-report [10, 36].

## Conclusion

Reasons for referral, as well as communication and coordination of SSc care are not yet properly aligned between rheumatologists and health professionals and tuned to the patients’ needs. Despite the high outcome satisfaction and the good accessibility of occupational therapists, psychologists, social workers, and hand therapists who are skilled to target unmet care needs such as psychological wellbeing, fatigue, daily functioning, and self-management, patients report relatively low utilization of health professional treatments. Our results suggest obstacles in the access to non-pharmacological care and barriers in communication between different (non-)pharmacological professionals. We recommend the development of easily accessible information and decision aids that give SSc patients and rheumatologists insights into the spectrum of non-pharmacological interventions and support the decision making for targeted referrals.

## Data Availability

The datasets used and/or analysed during the current study are available from the corresponding author on reasonable request.

## Abbreviations

SSc: Systemic sclerosis
HP: Health professional
ICF: International Classification of Function and Disability
HRQoL: Health-related quality of life
EULAR: European League Against Rheumatism
ARCH: Arthritis Research and Collaboration Hub
STROBE: Strengthening the Reporting of Observational Studies in Epidemiology
Castor EDC: Castor Electronic Data Capture
CQI: Consumer Quality Index
